# Identification of novel pathways and immune profiles related to sarcopenia

**DOI:** 10.3389/fmed.2023.928285

**Published:** 2023-04-17

**Authors:** Zeinab Abdelrahman, Xiaosheng Wang, Daming Wang, Tianfang Zhang, Yue Zhang, Xuhua Wang, Zuobing Chen

**Affiliations:** ^1^Department of Neurobiology and Department of Rehabilitation Medicine, First Affiliated Hospital, Zhejiang University School of Medicine, Hangzhou, Zhejiang, China; ^2^NHC and CAMS Key Laboratory of Medical Neurobiology, MOE Frontier Science Center for Brain Research and Brain–Machine Integration, School of Brain Science and Brain Medicine, Zhejiang University, Hangzhou, Zhejiang, China; ^3^Department of Neurobiology and Department of Orthopedics, Zhejiang University School of Medicine, 2nd Affiliated Hospital, Hangzhou, Zhejiang, China; ^4^Biomedical Informatics Research Lab, School of Basic Medicine and Clinical Pharmacy, China Pharmaceutical University, Nanjing, China; ^5^Big Data Research Institute, China Pharmaceutical University, Nanjing, China; ^6^Shenzhen Futian Hospital for Rheumatic Diseases, Shenzhen, China; ^7^Co-innovation Center of Neuroregeneration, Nantong University, Nantong, Jiangsu, China

**Keywords:** sarcopenia, low muscle mass, low physical performance, immunology, pathway analysis, bioinformatics, machine learning

## Abstract

**Introduction:**

Sarcopenia is a progressive deterioration of skeletal muscle mass strength and function.

**Methods:**

To uncover the underlying cellular and biological mechanisms, we studied the association between sarcopenia's three stages and the patient's ethnicity, identified a gene regulatory network based on motif enrichment in the upregulated gene set of sarcopenia, and compared the immunological landscape among sarcopenia stages.

**Results:**

We found that sarcopenia (S) was associated with GnRH, neurotrophin, Rap1, Ras, and p53 signaling pathways. Low muscle mass (LMM) patients showed activated pathways of VEGF signaling, B-cell receptor signaling, ErbB signaling, and T-cell receptor signaling. Low muscle mass and physical performance (LMM_LP) patients showed lower enrichment scores in B-cell receptor signaling, apoptosis, HIF-1 signaling, and the adaptive immune response pathways. Five common genes among DEGs and the elastic net regression model, *TTC39DP, SLURP1, LCE1C, PTCD2P1*, and *OR7E109P*, were expressed between S patients and healthy controls. *SLURP1* and *LCE1C* showed the highest expression levels among sarcopenic Chinese descent than Caucasians and Afro-Caribbeans. Gene regulatory analysis of top upregulated genes in S patients yielded a top-scoring regulon containing GATA1, GATA2, and GATA3 as master regulators and nine predicted direct target genes. Two genes were associated with locomotion: *POSTN* and *SLURP1*. *TTC39DP* upregulation was associated with a better prognosis and stronger immune profile in S patients. The upregulation of *SLURP1* and *LCE1C* was associated with a worse prognosis and weaker immune profile.

**Conclusion:**

This study provides new insight into sarcopenia's cellular and immunological prospects and evaluates the age and sarcopenia-related modifications of skeletal muscle.

## 1. Introduction

Sarcopenia is a syndrome characterized by a progressive deterioration of the skeletal muscle mass strength and function with a risk of developing adverse outcomes, such as motor disability and death (1). The study of sarcopenia and the development of preventive or therapeutic approaches represent public health priorities. The European Working Group on Sarcopenia in Older People (EWGSOP) recommends categorizing sarcopenia into three groups, namely pre-sarcopenia, sarcopenia, and severe sarcopenia (1). They proposed that the pre-sarcopenia stage is characterized by low muscle mass with no impact on muscle strength or physical performance, whereas the sarcopenia stage is characterized by low muscle mass with low muscle strength or low physical performance, and severe sarcopenia is characterized by the presence of all three criteria (1). Low physical performance is defined as a gait speed of <0.8 m/s, while low muscle strength is defined by handgrip strength of <30 kg for men and <20 kg for women (1). Low muscle mass is measured by a skeletal muscle mass index from 7.23 to 8.87 kg/m^2^ in men and from 5.45 to 6.42 kg/m^2^ in women (2). The pathophysiology of sarcopenia stages is still complex and partially characterized. There is an inadequate understanding of the underlying cellular and biological mechanisms driving the development of this disease. Till today, the current understanding of the regulation of muscle mass is mainly derived from animal studies data, with limited knowledge of the key regulatory processes in human muscle or blood. Animal studies suggested that the PI3k-Akt pathway is strongly linked to muscle synthesis (3). Most studies on sarcopenia in humans have suggested that a reduced synthetic response primarily drives the loss of muscle mass termed as anabolic resistance (4, 5).

The decline in immune function with age, known as immunosenescence, has been well-documented (6, 7). Immunosenescence includes inflammaging, which is the presence of a chronic proinflammatory state with age (8). Inflammaging is characterized by increased levels of proinflammatory cytokines, such as interleukin 1β (IL1β), interleukin 6 (IL6), tissue necrosis factor-alpha (TNFα), C-reactive protein (CRP), and a reduced level of anti-inflammatory cytokines, such as interleukin 10 (IL10) (8). Investigation of the immune system's role in different sarcopenia stages shows that the immune system's dysregulation may play a role in disease progression (8).

The current knowledge on the available sarcopenia biomarkers, such as functional, biological, or imaging-related biomarkers, that could be utilized in clinical trials remains inadequate. This study aimed to uncover the underlying cellular and biological mechanisms driving the development of sarcopenia and identify novel pathways and biomarkers that differentiate between different sarcopenia stages using unsupervised and supervised machine learning methods. Then, we studied the association of sarcopenia's three stages with the patient's ethnicity. In addition, we identified gene regulatory network mapping based on motif enrichment in the upregulated gene set of sarcopenia and identified the gene modules of coexpressed genes among the three sarcopenia stages. Finally, we compared the immunological landscape among sarcopenia stages based on their gene expression profiles. This study aimed to provide new insight into sarcopenia's cellular and immunological prospects, which is considered reliable and promising to evaluate the age and sarcopenia-related modifications of skeletal muscle.

## 2. Methods

### 2.1. Datasets

We downloaded the RNA-Seq gene expression in human muscle biopsies of 119 older adults (GSE111017) from the Gene Expression Omnibus (GEO) (https://www.ncbi.nlm.nih.gov/geo/). This dataset includes three individual datasets: Hertfordshire Sarcopenia, Jamaica Sarcopenia, and Singapore Sarcopenia. Hertfordshire's Caucasian dataset includes four sarcopenia patients with low muscle strength and physical performance, five with low muscle mass strength, three with low physical performance, and 28 healthy individuals (aged 68–77 years). The inclusion criteria of the Hertfordshire Caucasian dataset were the availability of birth records detailing birth weight, and men were excluded if they had heart disease, myositis, neuromuscular conditions, or diabetes. The diagnosis of sarcopenia among Caucasians was based on the EWGSOP algorithm (1, 9). Jamaica's Afro-Caribbean dataset includes nine sarcopenia patients with low muscle strength and physical performance, five with low muscle mass strength, 11 with low physical performance, and 14 healthy individuals, and all participants were of at least three grandparents of African origin (aged 63–89 years) (9). The diagnosis of sarcopenia among Afro-Caribbeans was also based on the EWGSOP definition (1). Singapore's Chinese dataset includes 20 sarcopenia patients and 20 healthy individuals without information on low muscle mass strength and the physical performance of older adults (aged 65–79 years). Each Chinese participant gave written self-reported ethnicity, and the diagnosis of sarcopenia was based on the AWGSOP definition (10). Lean mass was measured using DXA scanning (9). We merged the three datasets using the “merge” function in the R package “base”, performed batch effects adjustment, and normalized the combined data for further analyses.

### 2.2. Quantification of the enrichment levels of immune signatures

We used the single-sample gene-set enrichment analysis (ssGSEA) score (11) to evaluate the enrichment level of an immune signature in older adults based on the expression profiles of its marker genes. The ssGSEA score represents the enrichment score of a gene set in a sample based on the degree to which the gene set is coordinately upregulated/downregulated in the sample. We analyzed 30 immune signatures, including anti-inflammatory cytokines, B cells, CD4^+^ Regulatory T cells, CD8^+^ T cells, central memory T cells, cytolytic activity, cytolytic protein perforin, dendritic cells, effector memory T cell, effector T cell, effector Treg T cells, exhausted T cell, granulocyte, IGF1, immune-modulatory molecules, macrophages, MHC Class I, myokines, naive T cell, NK antimicrobial protein granulysin, NK encoded activating receptors, NK encoded inhibitory receptors, proinflammatory cytokines, resident memory T cell, resting Treg T cells, senescence-associated secretory phenotype (SASP), *Th1* cells, *Th2* cells, Type I IFN response, and Type II IFN response. The gene sets representing these immune signatures are listed in Supplementary material S1.

### 2.3. Pathway analysis

We identified differentially expressed genes (DEGs) between sarcopenia (S) and non-sarcopenia, low muscle mass (LMM)/non, and low physical performance (LMM_LP)/non using Student's *t*-test with a threshold of adjusted *P*-value [false discovery rate (FDR)] of <0.05 and fold change (FC) of mean expression levels of >1.3. Based on the DEGs, we identified KEGG (12) and GO (13) pathways differentially enriched between the identified groups using the “pathfindR” R package (14), with a threshold of FDR <0.05. The FDR was calculated by using the Bonferroni method (15).

### 2.4. Class prediction

We predicted sarcopenia (S) patients vs. non-sarcopenia control individuals based on the combined gene expression profiles. We performed 3-fold cross-validation (CV) in 33 sarcopenia patients and 86 control individuals. We trained the elastic net regression model classifier and predicted sarcopenia patients vs. non-sarcopenia control individuals. We used the CV to select the best λ with a fixed α parameter of 0.5. We reported the prediction performance (accuracy, specificity, balanced accuracy, and the Kappa statistic) in the 3-fold CV. We carried out the class prediction algorithm in the “caret” R package (16).

### 2.5. Gene regulatory and weighted correlation networks

We used iRegulon software (17) to directly enable gene regulatory network mapping based on motif enrichment in the upregulated gene set of S patients. The library of motifs used was 10K [9,713 position weight matrices (PWMs)], track collection of 1,120 ChiP-seq tracks, the putative regulatory region of 20 kb centered around transcription start site (TSS), motif ranking database 20 kb centered around TSS (seven species), and track ranking database of 20 kb centered around TSS (ChiP-seq-derived). In addition, we used an enrichment score threshold of 3.0, a ROC threshold for AUC calculation of 0.03, and a rank threshold of 5,000. For transcription factor (TF) prediction, the maximum FDR on the motif similarity threshold was 0.001. The weighted gene coexpression network analysis (WGCNA) was used to identify the gene modules of coexpressed genes among the three sarcopenia stages (LMM, LMM_LP, and S). Based on the expression correlations between the hub genes in gene modules, we identified the enriched GO pathways that have significant correlations with specific traits. We analyzed the WGCNA using the R package “WGCNA” (18).

### 2.6. Statistical analysis

We used Student's *t*-tests (two-tailed) to compare two classes of samples. In comparisons among three classes of samples, we used ANOVA tests (two-tailed). In evaluating correlations between gene expression levels and immune signatures' enrichment levels and between expression correlations between two genes, we used Pearson's correlation coefficients (*R*). We employed FDR to adjust *P*-values in multiple tests. The FDR was calculated using the Benjamini–Hochberg methods. We used abbreviations of S, LMM, and LMM_LP to represent sarcopenia, low muscle mass, and low muscle mass with low physical performance but without sarcopenia, respectively.

## 3. Results

### 3.1. Comparison of blood transcriptomes between sarcopenia, low muscle strength, and low physical performance patients with healthy individuals

We first identified the transcriptional signature of S patients compared to healthy individuals. This analysis revealed significant expression differences in sarcopenia patients in 459 unique protein-coding transcripts (419 upregulated and 40 downregulated genes, Supplementary material S1). In LMM patients, we found 74 unique protein-coding transcripts (60 upregulated and 14 downregulated genes, Supplementary material S1). In LMM_LP patients, we found 27 unique protein-coding transcripts (20 upregulated and seven downregulated genes, Supplementary material S1). There were five common genes among the three sets of DEGs, including *AC092184.1* (uncategorized gene); *FAM239B* (family with sequence similarity 239 member B pseudogene); *HSFX2* (heat shock transcription factor family, X-linked 2); *PTCD2P1* (pentatricopeptide repeat domain 2 pseudogene 1); and *SLURP1* (secreted LY6/PLAUR domain containing 1).

**Figure 1:**
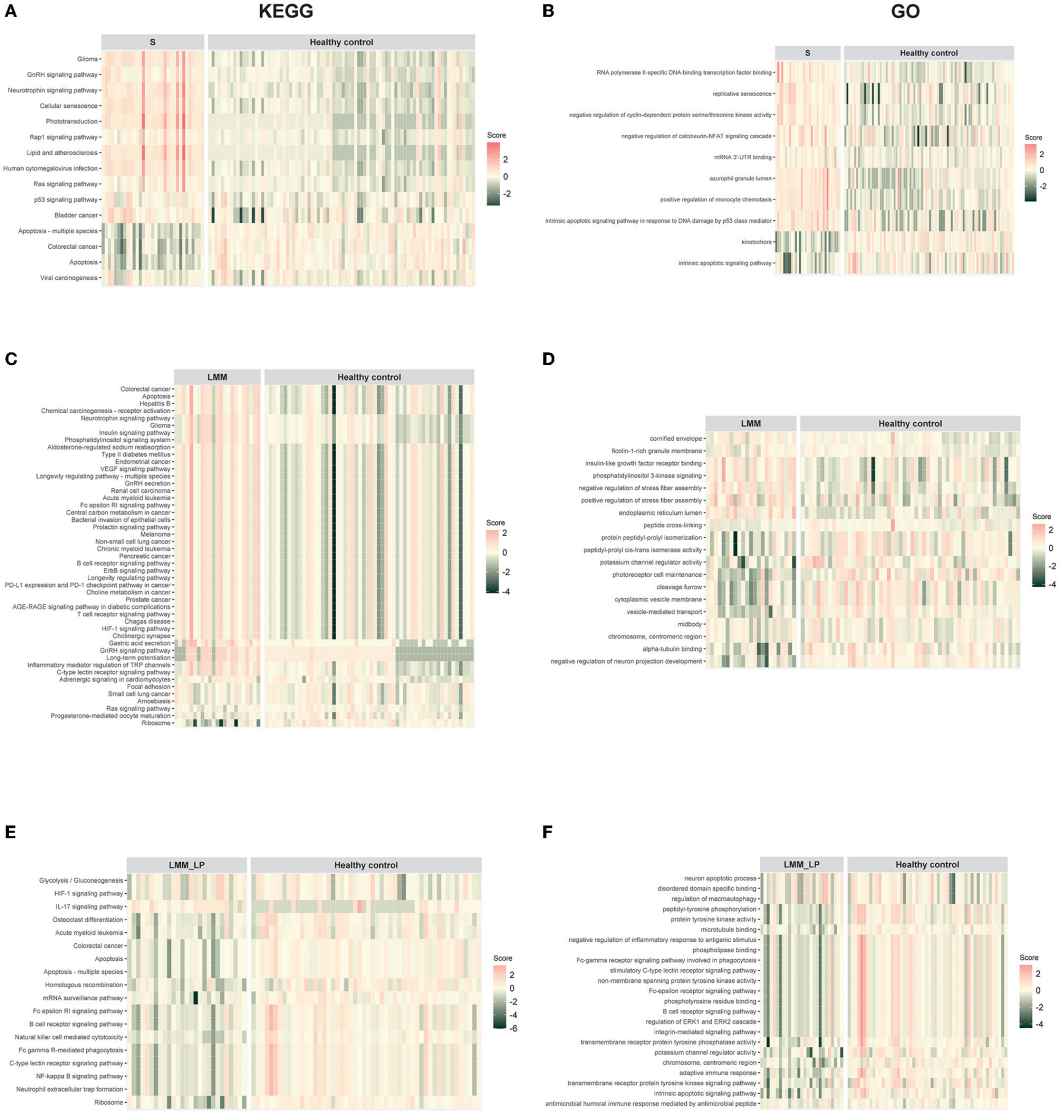
Pathway enrichment analysis for sarcopenia (S), low muscle mass (LMM), and low muscle mass with physical performance (LMM_LP) by unsupervised hierarchical clustering. **(A)** KEGG and **(B)** GO pathways enrichment analysis for sarcopenia (S) vs. healthy controls. **(C)** KEGG and **(D)** GO pathways enrichment analysis for low muscle mass (LMM) vs. healthy controls. **(E)** KEGG and **(F)** GO pathways enrichment analysis for low muscle mass with physical performance (LMM_LP) vs. healthy controls. The enrichment agglomerated z-score was calculated for each enriched term per sample.

We analyzed pathway enrichment on significant genes to identify activated or repressed pathways in a given sample or group. We calculated the agglomerated z-score of each enriched pathway per sample, followed by unsupervised hierarchical clustering of the most significant KEGG and GO pathways between the pre-defined groups. We found that sarcopenia was associated with increased expression of genes related to the GnRH, neurotrophin, Rap1, Ras, and p53 signaling pathways (Figure 1A). In addition, we found that sarcopenia patients showed significant enrichment in RNA polymerase II-specific DNA-binding transcription factor binding and negative regulation of cyclin-dependent protein serine/threonine kinase activity pathways (Figure 1B). Furthermore, the significantly activated pathways in LMM patients included the VEGF, B-cell receptor, ErbB, and T-cell receptor signaling pathways (Figures 1C, D). Interestingly, LMM_LP patients showed lower enrichment scores in the B-cell receptor signaling, apoptosis, HIF-1 signaling, and the adaptive immune response pathways than LMM patients (Figures 1E, F). These results indicate that immune responses and biological and metabolic dysregulation occurred simultaneously with the patient's muscle mass and function deterioration.

### 3.2. Application of elastic net regression model with feature selection to discriminate sarcopenia from healthy states

**Figure 2:**
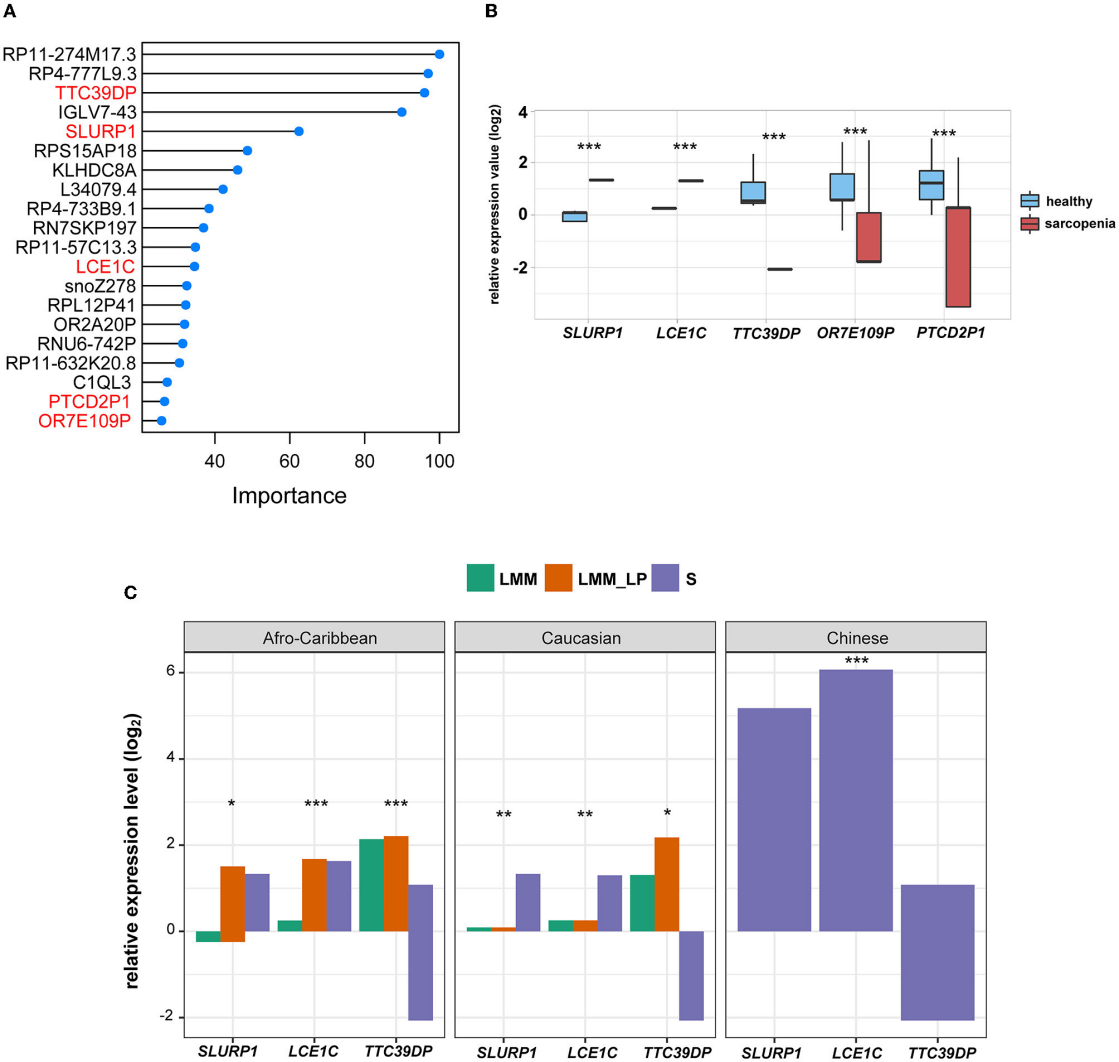
Discrimination of sarcopenia from healthy states using elastic net regression model and the association of the novel biomarkers with patient's ethnicity. **(A)** The elastic regression model discriminates sarcopenia from a healthy control based on gene expression profile. Model importance was identified as regression coefficients. **(B)** Comparison of the expression levels of the top-ranked genes between sarcopenia and the healthy controls between differentially expressed gene analysis and the elastic net regression model (*t*-test, *p*-values are shown. *p < 0.5, **p < 0.01, and ***p < 0.001). **(C)** Comparison of the expression levels of *SLURP1, LCE1C*, and *TTC39DP* among the S, LMM, and LMM_LP groups (ANOVA test, p-values are shown. *p < 0.5, **p < 0.01, and ***p < 0.00).

To identify the fewest transcripts as diagnostic biomarkers for S patients, we used the elastic net regression to derive a discriminating model in the transcriptomic data from S patients and healthy controls. We trained this model on multiple random samples of the transcriptomic data using CV and ranked all the genes based on their regression coefficients (Figure 2A). Among deferentially expressed genes (DEGs) and the elastic net regression model, we found five common genes (*TTC39DP, SLURP1, LCE1C, PTCD2P1*, and *OR7E109P*) that discriminated between sarcopenia patients and healthy controls with a different range (Figure 2A). The relative expressions of these genes were generally upregulated or downregulated in sarcopenia patients than in healthy controls (Figure 2B). To alleviate sampling error, we performed 3-fold CV train/test sequences to obtain an average accuracy of 0.97, 95% CI of 0.8871 and 0.9995, kappa of 0.95, a sensitivity of 0.97, a specificity of 1.00, and a balanced accuracy of 0.98. Among these top-ranked genes, we found that *SLURP1* and *LCE1C* were upregulated in S patients, while *TTC39DP, OR7E109P*, and *PTCD2P1* were downregulated in them (Figure 2B). *TTC39DP* (tetratricopeptide repeat domain 39D, pseudogene) was the highest ranked among the five genes and discriminated between S patients and healthy controls with a regression coefficient of 95.99. *SLURP1* and *LCE1C* discriminated between S patients and healthy controls with regression coefficients of 62.45 and 34.54, respectively (Figure 2A).

We further analyzed the expression differences of *TTC39DP, SLURP1*, and *LCE1C* in S, LMM, and LMM_LP among Caucasian, Afro-Caribbean, and Chinese descents (Figure 2C). *SLURP1* and *LCE1C* showed the highest expression levels in sarcopenic Chinese descent. In addition, *SLURP1* expression levels were higher in the S group of Caucasian descent than in the LMM and LMM_LP groups. *SLURP1* expression levels were higher in the LMM_LP group of Afro-Caribbean descent than in the S and LMM groups. *TTC39DP* showed the maximum expression levels among the LMM_LP group of Caucasian and Afro-Caribbean descent. These results indicate that different expression levels of *TTC39DP, SLURP1*, and *LCE1C* among the pre-defined groups depend on the patient's ethnicity.

### 3.3. Gene regulatory and weighted correlation networks of sarcopenia, low muscle mass, and low physical performance patients

**Figure 3:**
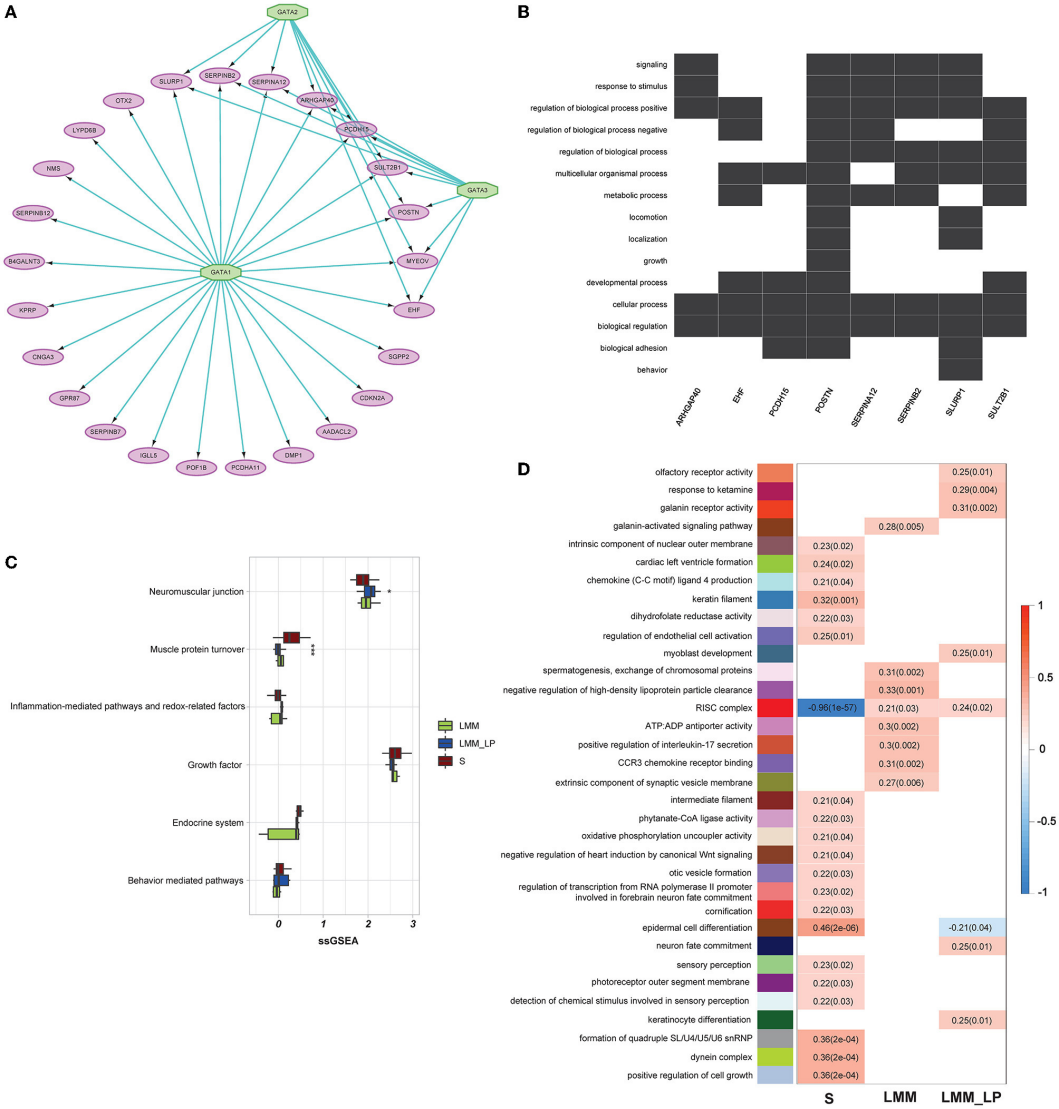
Gene regulatory, weighted correlation networks, and pathophysiological mechanisms of different sarcopenia stages, low muscle mass, and low physical performance patients. **(A)** Gene regulatory network of the top upregulated genes under sarcopenia using iRegulon software. Results show GATA1, 2, and 3 as top regulators and their relationship with the nine predicted direct targets. **(B)** Heatmap-like functional classification depicts the linkages of predicted genes and biological concepts (e.g., GO terms or KEGG pathways). **(C)** Comparison of the pathophysiological mechanisms between the S, LMM, and LMM_LP groups (ANOVA test, *p*-values are shown. *p < 0.5, **p < 0.01, and ***p < 0.00). **(D)** Significant gene modules and their representative gene ontology differentially enriched between sarcopenia (S), low muscle mass (LMM), and low muscle mass with physical performance (LMM_LP), which were identified by WGCNA. The *P*-values are shown in parentheses.

We analyzed 96 significantly upregulated genes under S status using iRegulon software. The analysis yields a top-scoring regulon containing GATA1, GATA2, and GATA3 as master regulators and nine predicted direct target genes, namely *EHF, SLURP1, MYEOV, SERPINB2, SERPINA12, ARHGAP40, PCDH15, SULT2B1*, and *POSTN* (Figure 3A). The predicted GATA1, GATA2, and GATA3 targets are likely functional targets associated with the different pathways, including signaling, response to stimulus, regulation of the biological process, multicellular organismal process, metabolic process, locomotion, localization, growth, developmental process, cellular process, biological regulation, and biological adhesion behavior (Figure 3B). Interestingly, two genes were associated with locomotion: *POSTN* and *SLURP1*. *POSTN* has been associated with metastatic outgrowth in melanoma (19), while the association of *SLURP1* with locomotion remains limited.

Furthermore, we conducted further analysis to investigate biomarkers of different sarcopenia stages, according to different pathophysiologic mechanisms. These mechanisms include the neuromuscular junction, endocrine system, growth factor, muscle protein turnover, behavior-mediated pathways, inflammation-mediated pathways, and redox-related factors (20). However, due to the small sample size, significant results were found only in muscle protein turnover and neuromuscular junction pathways (Figure 3C). As expected, muscle protein turnover and neuromuscular junction pathways were higher and lower with severe sarcopenic patients than with LMM and LMM_LP, respectively. These results indicate the association of sarcopenia with the impairment of neurophysiological functions, alteration in the transduction of the action potentials of muscle, and structural alterations of the muscles.

In addition, WGCNA identified 34 significant gene modules differentially enriched between the S, LMM, and LMM_LP groups (Figure 3D). The 34 representative colors of these modules are indicated in salmon2, maroon, orangered1, sienna4, lightpink4, yellowgreen, paleturquoise, steelblue, lavenderblush2, lightslateblue, skyblue4, thistle1, mediumorchid, red, violet, coral3, mediumpurple3, yellow4, indianred4, plum, antiquewhite2, mediumpurple4, salmon4, mediumpurple2, lightcoral, brown2, saddle brown, lightgreen, darkmagenta, lightcyan, darkgreen, grey60, greenyellow, and lightsteelblue. The representative GO terms for these gene modules were olfactory receptor activity, response to ketamine, galanin receptor activity, galanin-activated signaling pathway, intrinsic component of nuclear outer membrane, cardiac left ventricular formation, chemokine (C-C motif) ligand 4 production, keratin filament, dihydrofolate reductase activity, regulation of endothelial cell activation, myoblast development, spermatogenesis exchange of chromosomal protein, negative regulation of high-density lipoprotein particle clearance, RISC complex, ATP:ADP antiporter activity, positive regulation of interleukin-17 secretion, CCR3 chemokine receptor binding, extrinsic component of synaptic vesicle membrane, intermediate filament, phytanate-CoA ligase activity, oxidative phosphorylation uncoupler activity, negative regulation of heart induction by canonical Wnt signaling, otic vesicle formation, regulation of transcription from RNA polymerase II promoter involved in forebrain neuron fate commitment, cornification, epidermal cell differentiation, neuron fate commitment, sensory perception, photoreceptor outer segment membrane, detection of chemical stimulus involved in sensory perception, keratinocyte differentiation, formation of quadruple SL/U4/U5/U6 snRNP, dynein complex, and positive regulation of cell growth, respectively.

The S group was associated with enriched pathways of intrinsic component of the nuclear outer membrane, cardiac left ventricle formation, chemokine (C-C motif) ligand 4 production, keratin filament, dihydrofolate reductase activity, regulation of endothelial cell activation, intermediate filament, phytanate-CoA ligase activity, oxidative phosphorylation uncoupler activity, negative regulation of heart induction by canonical Wnt signaling, otic vesicle formation, regulation of transcription from RNA polymerase II promoter involved in forebrain neuron fate commitment, cornification, epidermal cell differentiation, sensory perception, photoreceptor outer segment membrane, detection of chemical stimulus involved in sensory perception, formation of quadruple SL/U4/U5/U6 snRNP, dynein complex, and positive regulation of cell growth (Figure 3D). In contrast, the red RISC complex module was negatively associated with the S group. Moreover, the LMM and LMM_LP groups shared two common pathways with the S group, namely epidermal cell differentiation and the RISC complex. The RISC complex pathway was highly enriched in the LMM and LMM_LP groups, while the S group observed the opposite. Interfering RNAs (siRNAs) and microRNAs (miRNAs) act *via* RNA-induced silencing complexes (RISC) and can regulate gene transcripts negatively (21). RISC involves many biological processes, such as brain aging (21). It has been proven that the dysregulation of miRNA expression has been associated with reduced muscle plasticity (22), as observed in our results, and, as a consequence, will impair skeletal muscle adaptations to exercise (22).

### 3.4. The immune profile of sarcopenia, low muscle mass, and low physical performance patients

**Figure 4:**
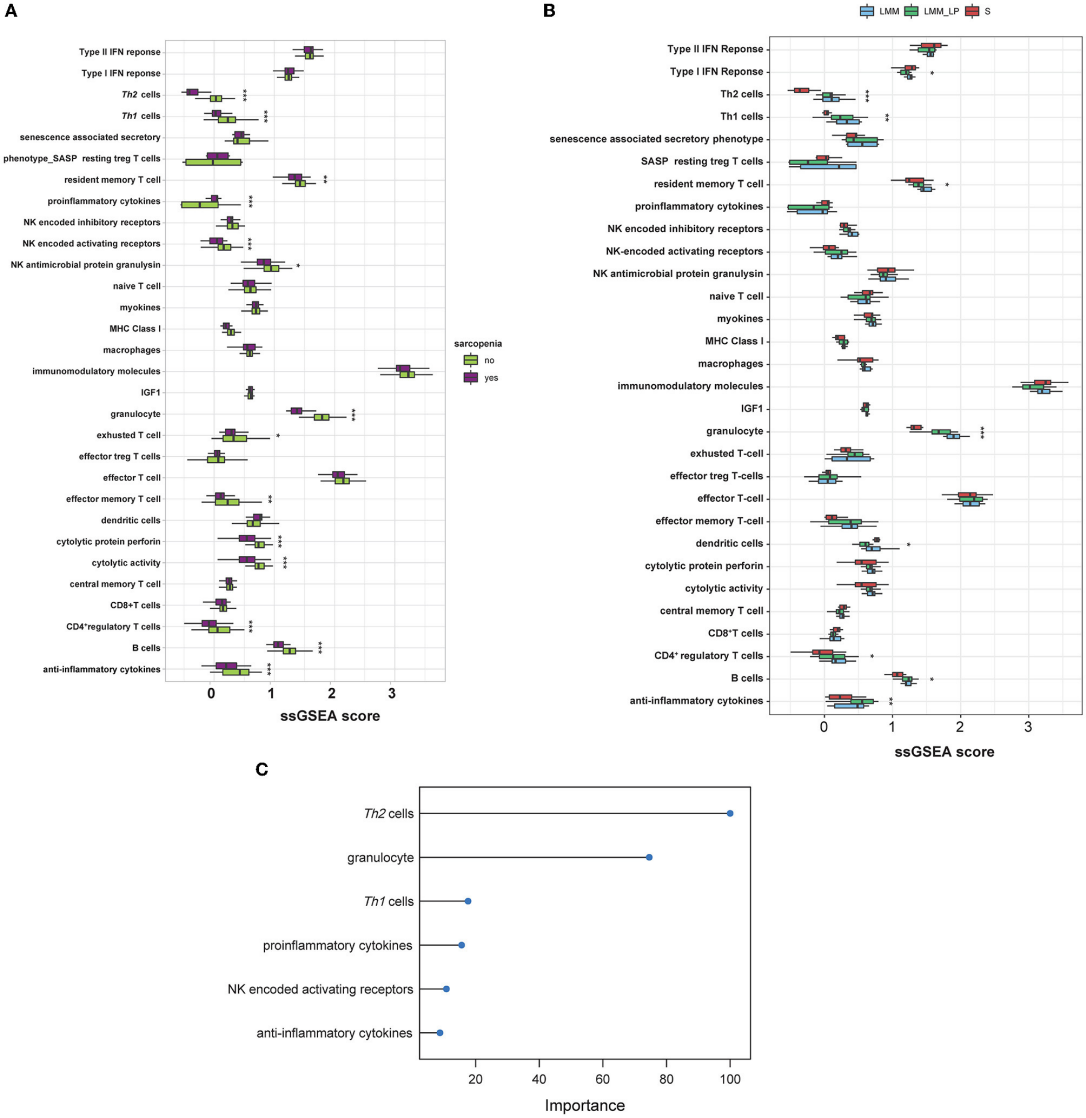
Immunological landscape of the S, LMM, and LMM_LP groups. **(A)** Comparison of 30 immune signature expression levels between sarcopenia and healthy controls (*t*-test, *p*-values are shown. *p < 0.5, **p < 0.01, and ***p < 0.00). **(B)** Comparison of 30 immune signature expression levels between the S, LMM, and LMM_LP groups (ANOVA test, *p*-values are shown. *p < 0.5, **p < 0.01, and ***p < 0.00). **(C)** The elastic regression model discriminates sarcopenia from a healthy control based on 30 immune signature expression profiles. Model importance was identified as regression coefficients.

We analyzed the association of different immune signatures with patients' muscle mass and function deterioration. First, we conducted a *t*-test analysis to identify the immune signatures (30 immune signatures) differentiating sarcopenia from healthy individuals. A total of 14 immune signatures significantly differentiated sarcopenia from healthy individuals (Figure 4A). Only proinflammatory cytokines were significantly upregulated in sarcopenia than in healthy controls. In contrast, Th1&Th2 cells, resident memory T cells, NK activating receptor, NK antimicrobial protein granulysin, granulocytes, exhausted T cells, effector memory T cells, cytolytic protein perforin, cytolytic activity, CD4^+^ T cells, B cells, and anti-inflammatory cytokines were less enriched in sarcopenia patients than in healthy controls. Second, we used the ANOVA test to compare immune signatures' expression levels among the S, LMM, and LMM_LP groups. In total, nine immune signatures significantly differentiate the three groups (Figure 4B). Type I IFN response and dendritic cells were higher in sarcopenia patients than in the other two groups. However, signatures of Th1&Th2 cells, resident memory T cells, granulocytes, CD4^+^ T cells, B cells, and anti-inflammatory cytokines declined with worsening muscle function and disease progression. These results strongly correlate immune system dysregulation with disease progression and muscle function deterioration.

Furthermore, we used the elastic net regression to derive a discriminating model of the 30 immune signatures based on patients with and without sarcopenia after training this model on multiple random samples of the immune signatures using CV. We ranked all the immune signatures based on their regression coefficients. We found that Th2 cells and granulocytes could discriminate between sarcopenia and healthy individuals with a regression coefficient of 100 and 77.9, respectively (Figure 4C). Figures 4A, B show that the Th2 cells were significantly downregulated in sarcopenia patients relative to healthy controls, LMM, and LMM_LP. These results indicate that sarcopenia patients have weaker immune responses than other pre-defined groups.

### 3.5. Association of *TTC39DP, SLURP1*, and *LCE1C* expression levels with immune signatures

**Figure 5:**
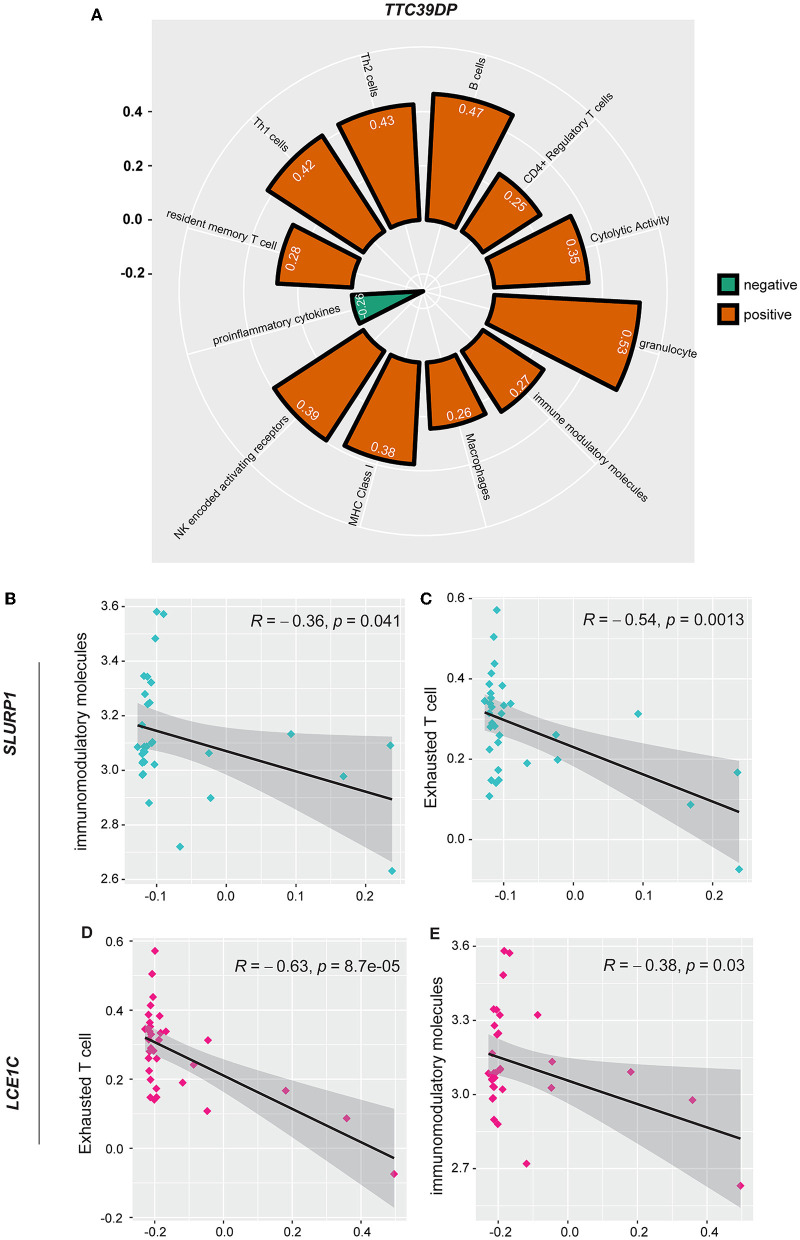
Association between *SLURP1, LCE1C*, and *TTC39DP* expression levels and immune signatures. **(A)** Significant correlation between *TTC39DP* expression levels and 12 immune signatures. **(B)** Significant correlation between *SLURP1* expression levels with immunomodulatory molecules and **(C)** exhausted T cell. **(D)** Significant correlation between *LCE1C* expression levels with an exhausted T cell. **(E)** Immunomodulatory molecules. Pearson's correlation coefficients (*R*) and *P*-values are shown.

In addition, we analyzed the association of various immune signatures with the pre-defined top-ranked genes (*TTC39DP, SLURP1*, and *LCE1C*) that discriminated sarcopenia from healthy individuals. *TTC39DP* has shown a significant positive correlation with 11 immune signatures and one negative correlation with proinflammatory cytokines (Figure 5A). The highest positive correlations were observed in granulocytes and B cells. In contrast, *SLURP1* and *LCE1C* significantly negatively correlated with immunomodulatory molecules and exhausted T cells (Figures 5B–E). Interestingly, *TTC39DP* expression levels were much lower in sarcopenia patients than in healthy controls, and its upregulation correlated with a better prognosis and stronger immune profile in older people. In contrast, *SLURP1* and *LCE1C* expression levels were much higher in sarcopenia patients and associated with a worse prognosis and weaker immune profile.

### 3.6. Gene coexpression networks of *TTC39DP, SLURP1*, and *LCE1C*

**Figure 6:**
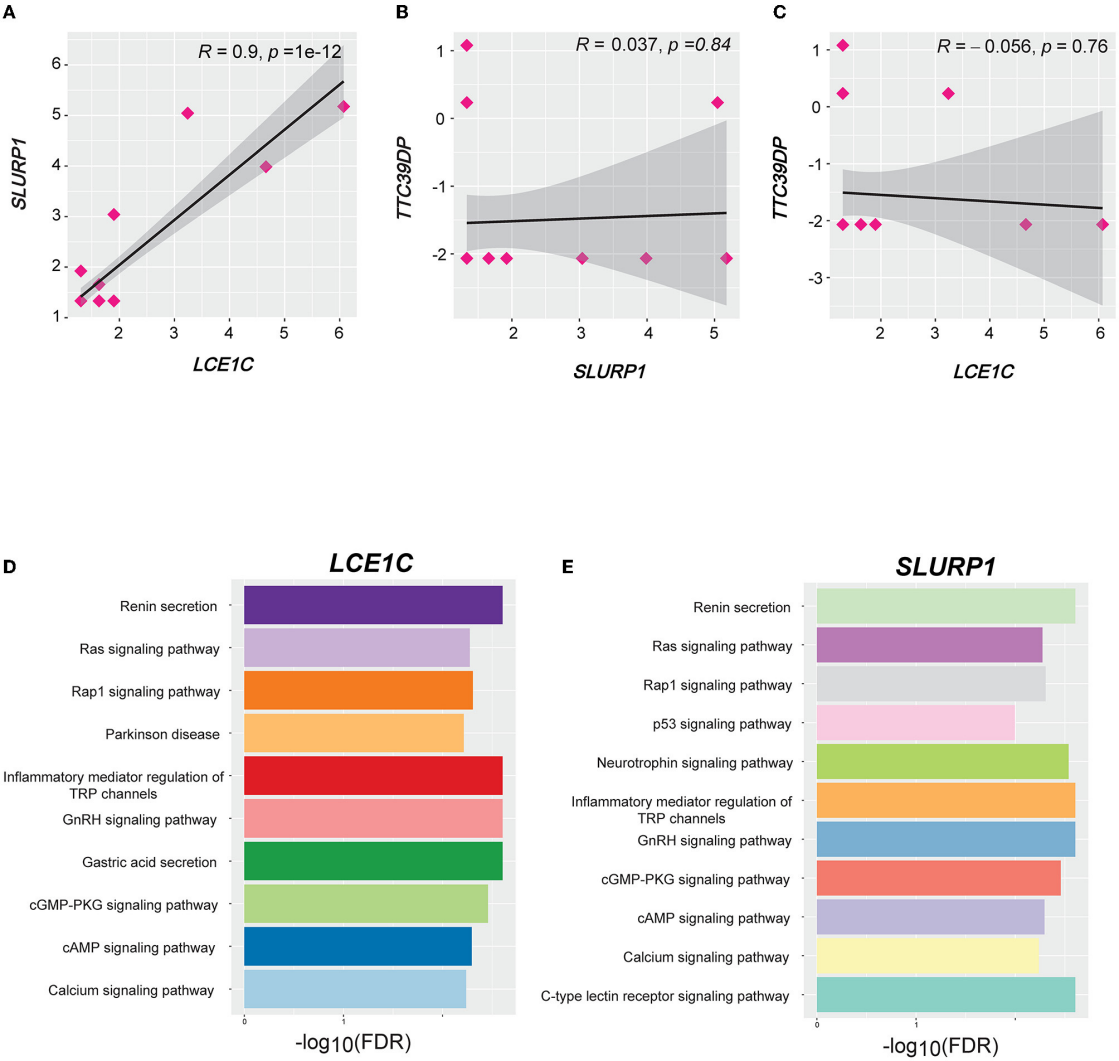
Correlation and pathways associated with *LCE1C, SLURP1*, and *TTC39DP* expression levels. **(A)** Correlation between *LCE1C* and *SLURP1* expression levels. **(B)** Correlation between *TTC39DP* with *SLURP1* and **(C)** *LCE1C* expression levels. Pearson's correlation coefficients (*R*) and *P*-values are shown. **(D)** The KEGG pathways enriched with *LCE1C* and **(E)** *SLURP1* highly correlated genes (*R* > 0.5) were identified by GSEA (53).

We identified 103, 57, and 66 genes with strong positive expression correlations with *TTC39DP, SLURP1*, and *LCE1C*, respectively (*R* > 0.5). Interestingly, *SLURP1* and *LCE1C* showed a strong positive expression correlation (*R* = 0.9, *p* < 0.001, Figure 6A). In contrast, no significant correlations existed between *SLURP1* and *LCE1C* with *TTC39DP* (Figures 6B, C). Pathway enrichment analysis identified several significant KEGG pathways associated with *SLURP1* and *LCE1C* expression, including Renin secretion, Ras signaling pathway, Rap1 signaling pathway, Parkinson's disease, inflammatory mediator regulation of TRP channels, GnRH signaling pathway, gastric acid secretion, cGMP-PKG signaling pathway, cAMP signaling pathway, calcium signaling pathway, p53 signaling pathway, neurotrophin signaling pathway, and C-type lectin receptor signaling pathway. These pathways were associated with the 57 and 66 genes strongly correlated with *SLURP1* and *LCE1C*, respectively (Figures 6D, E, FDR < 0.05). However, pathway enrichment analysis with 103 correlated genes with *TTC39DP* did not show significantly enriched pathways.

## 4. Discussion and conclusion

Sarcopenia is a progressive and generalized skeletal muscle disorder, including the accelerated loss of muscle mass function (23). However, identifying biomarkers that reflect the central pathogenic processes of sarcopenia has remained lacking. The main problem in the diagnosis of sarcopenia is its complex genesis. The pathophysiology of sarcopenia includes inflammatory conditions, endocrine dysfunctions, and glycogen, glucose, and lipid metabolism dysregulation (24). Similarly, muscle-related cytokines and myokines show endocrine actions between muscle and tissues. In addition, many factors related to chronic diseases and lifestyles, such as obesity and low physical activity, may define the development of sarcopenia (25). Regardless of hopeful advances in assessing muscle mass and strength, various mechanisms of sarcopenia have not been fully distinguished, although several biomarkers may be found in both blood and tissue samples (26). Even though histology is the primary standard for recognizing the pathological mechanisms of different sarcopenic stages, biopsy samples are often a complicated process and not acceptable to older adult patients, especially during the follow-up of sarcopenic patients. Therefore, the emerging importance is identifying potential biomarkers for sarcopenia. In this study, we attempted to provide promising biomarkers and pathways that can detect sarcopenia at its early stages.

We found that sarcopenia is associated with increased expression levels of genes related to the neurotrophin, Rap1, Ras, and p53 signaling pathways. Neurotrophin signaling is involved in synaptic plasticity, memory, and neuronal health (27). In addition to sarcopenia and neurotrophin signaling during aging, the accumulation of proinflammatory cytokines generates neurotrophin resistance, increasing cognitive decline and dementia risk (27). *In vivo* studies showed that mice deficient for the tissue-specific Ras signaling, *RasGrf1*, mainly expressed within the pancreatic islets and the hippocampus and hypothalamus regions of the brain, were long-lasting and exhibited better motor coordination in elder animals than in their control (28). In addition, human studies showed that Ras signaling activation is associated with premature aging, including osteoporosis and osteopenia (28). p53 signaling has also been shown to contribute to aging. Preclinical studies showed that naturally aged mice had longevity linked with diminished p53 function, and p53 activation or dysregulation can accelerate aging (29).

In addition, we found that LMM_LP patients showed lower enrichment scores in B-cell receptor signaling, apoptosis, HIF-1 signaling, and adaptive immune response pathways than LMM patients, indicating that immune response and metabolic and biological dysregulation occurred simultaneously with the patient's muscle mass and function deterioration. B cells are involved in the immunosuppressive regulatory process, and during aging, the depletion of the secretion of IL7 and IGF1 causes alterations in the number and function of B cells. Dysfunctional B cells were correlated with the dysregulation of immune aging, affecting the regeneration and strength of the skeletal muscle. It has been suggested that B-cell accumulation in tissues plays a role in sarcopenia obesity, which is related to skeletal muscle inflammation (30). HIF-1 is vital as a mediator in adaptation to hypoxia, which increases oxygen delivery and glucose transporters expression during hypoxia. During sarcopenia, HIF-1 impairs oxidative metabolism and mitochondrial biogenesis, causing muscle mass function deterioration (31).

Furthermore, we found five common genes, *TTC39DP, SLURP1, LCE1C, PTCD2P1*, and *OR7E109P*, that can differentiate between sarcopenia patients and healthy controls. *SLURP1* and *LCE1C* show the highest expression levels among sarcopenic Chinese descent than Caucasian and Afro-Caribbean. *TTC39DP* was the highest in LMM_LP than *SLURP1* and *LCE1C* in Afro-Caribbean and Caucasian descents. *SLURP1* is an immunomodulatory protein that promotes corneal immune and angiogenic processes (32). It is expressed in tissues such as skin, palms, soles, and oral and bronchial cells (32). *SLURP1* suppresses TNFα production from T cells and IL1β, IL6, IFNγ, and IL8 secretion, which mimic the anti-inflammatory effect (33). Although we found that proinflammatory cytokines were higher in sarcopenia patients than in healthy control, this could trigger *SLURP1* activation. *SLURP1* was also identified as an epidermal neuromodulator essential for epidermal homeostasis, which indicates this gene's crucial role in aging (34). *LCE1C* and *TTC39DP's* role in aging and sarcopenia development remains lacking, even though reports have identified *LCE1C* as essential in epithelial development (35) and the identification of the skin (36). Although the results report a strong correlation between *LCE1C* and aging, the correlation of this gene with sarcopenia and muscle function deterioration is still needed.

Gene regulatory analysis yields a top-scoring regulon containing GATA1, GATA2, and GATA3 as master regulators and nine predicted direct target genes, and these predicted targets were associated with different pathways. Among these nine targeted genes, *POSTN* and *SLURP1* showed strong associations with locomotion. *POSTN* (periostin) is an extracellular matrix protein crucial in myocardial fibrosis and heart inflammatory processes (37). Periostin was associated with heart aging, and excessive periostin expression contributed to cardiomyocyte senescent (37). In addition, periostin was found to have a maintenance role in muscle mass during muscle regeneration (38). Furthermore, upregulation and secretion of periostin increase muscular dystrophy in mice, and deletion of periostin led to the improvement in skeletal muscle structure and function, suggesting its importance in muscle strength and function (39). However, although our results showed an association of *SLURP1* with locomotion and disease progression, supporting data regarding the role of this gene in muscle function and sarcopenia remain lacking, and further studies are recommended.

As mentioned, sarcopenia is a disease of muscle constrictive dysfunction, metabolic abnormalities, and systemic inflammation (40). Thus, sarcopenia is a disease of more complex networks involving different pathophysiologic mechanisms from dysfunction of neuromuscular junctions, a decline of the endocrine system, imbalance of growth enhancer and suppressor factors, structural alteration of protein synthesis, behavior-mediated pathways (e.g., physical activity and obesity), and inflammation (20). Neuromuscular junction dysfunction could gradually alter muscle action potentials during exercise and be associated with neuromuscular weakness, as shown in Figure 3C (41). In addition, one of the earlier signs of sarcopenic damage would be the early structural alterations of the muscle protein. The essential biomarkers in the evaluation of muscle mass are serum sarcomeric proteins, such as actin, troponin, creatinine, and extracellular matrix proteins (42); in addition, type VI collagen (IC6), MMP-generated degradation fragment of collagen 6 (C6M) (43), the N-terminal peptide (P3NP) (44), and 3-methylhistidine (3MH) (45) are also associated with the pathophysiology of sarcopenia.

In addition, WGCNA analysis revealed that sarcopenia was associated with multiple enriched pathways and was negatively associated with the RISC complex. We also found that the LMM and LMM_LP groups shared two common pathways with the S group, epidermal cell differentiation, and the RISC complex. As observed in sarcopenia, the RISC complex pathway was associated with muscle mass deterioration and function. The RISC pathway involves many biological processes, such as brain aging (21). It has been proven that the dysregulation of miRNA expression (act *via* RISC complex) has been associated with reduced muscle plasticity (22) and will impair skeletal muscle adaptations to exercise (22).

From the immunological perspective, we found that proinflammatory cytokines, type I IFN response, and dendritic cells were significantly upregulated in sarcopenia patients. These cytokines are known to play a critical role in sarcopenia development by causing destructive effects on the skeletal muscle, declined physical performance and muscle strength, and disability in older adults (46). Similarly, inflammatory cytokines such as IL-1 can block the differentiation of myoblasts in the presence of overexpressed activin, and this synergic activity was confirmed in models related to sarcopenia (47). In addition, proinflammatory cytokines highlight the complexity of the inflammaging process. It was shown that patients suffering from obesity who develop sarcopenic obesity in older age show elevated levels of proinflammatory cytokines, indicating the strong association between endocrine, metabolic, and inflammaging (48).

In contrast, Th1&Th2 cells, resident memory T cells, NK activating receptor, NK antimicrobial protein granulysin, granulocytes, exhausted T cells, effector memory T cells, cytolytic protein perforin, cytolytic activity, CD4^+^ T cells, B cells, and anti-inflammatory cytokines were lower in sarcopenia patients than healthy controls, indicating the strong association of immune system dysregulation with disease progression (49). During immune aging, the reservoir of NK cells in the thymus is nearly depleted, and depleted NK cells were found to be associated with muscle loss in sarcopenia and mortality (50). Furthermore, dendritic cells are related to immune cells' maturation and differentiation, such as Th1 and Th2 cells, and develop an inflammatory environment during immune aging, affecting skeletal muscle regeneration (51). However, there is still a lack of knowledge on the particular role of each immune cell subtype in sarcopenia, but their whole function may work in the skeletal muscle environment modification and affect the physiological homeostasis of skeletal muscle (52).

This study has several limitations. First, because sarcopenia is a complex disease, obtaining biopsy samples is not usually acceptable to older adult patients, which is why patients' samples are relatively small. Second, we obtained the results by bioinformatics analyses without experimental justification, so further experimental and clinical investigations are required to assess these biomarkers' activity and beneficial effects. In conclusion, this study identified the association between immune responses and biological dysregulation with the patient's muscle mass and function deterioration. Three biomarkers were identified, *TTC39DP, SLURP1*, and *LCE1C*, and their expression levels depend on the patient's ethnicity and immune profile. *TTC39DP* was correlated with a better prognosis and stronger immune profile, while *SLURP1* and *LCE1C* were associated with a bad prognosis and weaker immune profile. In addition, a strong association between immune system dysregulation with disease progression and muscle function deterioration was observed. This study provides new insight into sarcopenia's cellular and immunological prospects, which is considered reliable and promising to evaluate the age and sarcopenia-related modifications of skeletal muscle.

## Data availability statement

Publicly available datasets were analyzed in this study. This data can be found here: https://www.ncbi.nlm.nih.gov/geo/query/acc.cgi?acc=GSE111017.

## Ethics statement

Ethics approval was waived since we used only publicly available data and materials in this study.

## Author contributions

ZA: conceptualization, methodology, software, validation, formal analysis, investigation, resources, data curation, writing—original draft, writing—review and editing, and visualization. XiW: conceptualization, methodology, software, validation, formal analysis, investigation, resources, data curation, writing—original draft, writing—reviewing and editing, visualization, and supervision. DW and TZ: formal analysis and investigation. YZ: methodology and investigation. XuW and ZC: conceptualization, methodology, investigation, writing—reviewing and editing, supervision, project administration, and funding acquisition. All authors contributed to the article and approved the submitted version.
